# Wrist-type home blood pressure monitoring device improves usability and preserves sleep quality compared with conventional ambulatory monitoring

**DOI:** 10.1097/HJH.0000000000004297

**Published:** 2026-03-20

**Authors:** Cornelia J.C. Vermeer, Carline J. Van den Dries, Monika Hollander, Geert-Jan Geersing, Frans H. Rutten

**Affiliations:** a Department of General Practice & Nursing Science, Julius Centre for Health Sciences and Primary Care, University Medical Centre Utrecht (UMCU), Utrecht University, Utrecht; b Department of Cardiovascular and Metabolic Health, Amsterdam University Medical Centre (AUMC), Amsterdam, The Netherlands

**Keywords:** home blood pressure measurement, nocturnal blood pressure, sleep disturbance, upper arm-cuff device, user-friendliness, wrist-cuff device

## Abstract

**Objective:**

To compare usability and impact on sleep quality of a wrist-type home blood pressure monitoring (HBPM) device and a conventional upper-arm 24-h ambulatory blood pressure monitoring (ABPM) device.

**Methods:**

In this randomized crossover study, 60 community-dwelling individuals aged 50–80 years from the Reviving Early Detection of cardiovascular disease in the Utrecht Health Project (RED-LRGP) trial underwent wrist-type HBPM (Omron HEM-9601T) and upper-arm ABPM. HBPM included three nocturnal measurements per night (4 h after bedtime, 02 : 00, and 04 : 00) for five nights, with additional morning and evening daytime measurements. ABPM included a single 24-h period with hourly cuff inflations. After each monitoring period, participants completed the system usability scale (SUS) and visual analogue scale (VAS) for sleep quality. Linear mixed-effects models were used to compare usability (SUS) and sleep quality (VAS) between devices.

**Results:**

Sixty participants (mean age 58 years, 57% female, 23% hypertensive) completed questionnaires. HBPM showed higher usability (SUS 71.9 versus 52.4; *P* < 0.0001) and better sleep quality (VAS 74.6 versus 58.9; *P* < 0.0001) than ABPM. With five nightly HBPM measurements, nocturnal hypertension was detected in 27–39% of participants. This was 26% using single-night ABPM measurement. Nondipping patterns occurred in 7–14% with HBPM versus 25% using ABPM. Sixteen participants without obesity, hypertension, or other relevant comorbidities (27%) were classified as HBPM nondippers on ≥1 night.

**Conclusions:**

Wrist-type HBPM, combining multinight automated monitoring with high usability and minimal sleep disruption represents a feasible, patient-centered tool for home-based nocturnal BP assessment that has the potential to capture night-to-night BP variability.

## INTRODUCTION

Hypertension is a major risk factor for morbidity and mortality and is strongly associated with cardiovascular disease (CVD) [1]. Beyond daytime measurements, nighttime blood pressure (BP) has emerged as an important predictor of CVD, particularly in the presence of nocturnal hypertension and nondipping patterns [3]. Accurate monitoring and control of BP are therefore essential for effective cardiovascular risk management.

Twenty-four-hour ambulatory blood pressure monitoring (ABPM) using validated upper-arm cuff devices is the established standard for assessing nocturnal BP and is recommended in current guidelines for hypertension management [2,4]. However, accumulating evidence indicates that nocturnal home blood pressure monitoring (HBPM) using validated upper-arm devices provides nighttime BP measurements comparable to those obtained by ABPM [5,6]. Moreover, upper-arm HBPM shows similar associations with hypertension-mediated target organ damage as ABPM [5], and nocturnal hypertension detected by HBPM is a significant predictor of future cardiovascular events [7–10]. During nocturnal ABPM, automatic BP measurements are obtained over a single night using a device preset by healthcare staff. In contrast, nocturnal HBPM requires patients to prepare the device before bedtime and remove it upon waking, usually over multiple nights, with several preset measurements taken automatically each night.

However, repeated cuff inflations with traditional upper-arm cuff BP devices may disturb sleep, potentially affecting the reproducibility and accuracy of nocturnal BP assessment [11]. To address these issues, the wrist-type HBPM device Omron HEM-9601T (NightView) was developed, incorporating a timer function and position-specific algorithms for automated nocturnal BP measurement. Validation studies have shown that NightView provides reliable self-measured BP values and is suitable for automatic measurements during sleep in the supine position [12,13]. However, the impact of wrist-type devices on sleep quality and their usability during self-measured nighttime BP monitoring has not yet been investigated.

Therefore, the present study aimed to compare the usability and impact on sleep quality of a wrist-type HBPM device and a conventional upper-arm 24-h ABPM device. Furthermore, we explored the agreement in classification between HBPM and ABPM for nocturnal hypertension and dipping patterns, using method-specific measurement schedules.

## METHODS

### Study design

The Night-LRGP study was a randomized crossover substudy nested within the intervention arm of the Reviving Early Detection of cardiovascular disease in the Utrecht Health Project (UHP; Dutch: Leidsche Rijn Gezondheidsproject, LRGP; RED-LRPG) study [14]. The Night-LRGP study evaluated usability, impact on sleep quality, and nocturnal BP patterns using wrist-type HBPM and upper-arm ABPM, with each participant undergoing both monitoring methods. The cross-over design allowed direct within-subject comparisons while minimizing inter-individual variability. The order of device use (HBPM first or ABPM first) was determined by nonblinded randomization, thereby minimizing potential ‘order effects’, such as the first device influencing the participant's experience with the second device.

### Study participants

Participants were drawn from the RED-LRPG study, a diagnostic randomized controlled trial in community-dwelling individuals aged 50–80 years. In the parent trial, participants were randomized to a screen-like early detection strategy (intervention arm) or usual care (control arm) to assess the yield of this strategy on incident coronary artery disease (CAD), atrial fibrillation (AF), heart failure (HF), and/or valvular disease (VHD). Only intervention-arm participants were included in this study to avoid contamination of the control arm of the RED-LRGP study. The main exclusion criterion in the RED-LRGP study was a confirmed triple diagnosis of CAD, AF and HF, since a strategy aimed at earlier detection of these conditions would have no benefit in such patients. Other key exclusion criteria included a terminal condition, severe cognitive impairment, or inability to provide informed consent. For the current Night-LRGP study, participants with known AF were additionally excluded as this condition hampers automatic BP measurement.

All participants provided written informed consent. The study was conducted in accordance with the principles of the Declaration of Helsinki and the trial protocol was approved by the Ethics Review Committee of University Medical Center Utrecht (NL82944.041.23).

### Outcomes

The primary outcomes were the usability and impact on sleep quality of a wrist-type HBPM device, used for 5 days and nights, compared with a single 24-h measurement period with a conventional upper-arm ABPM device. The secondary outcomes were the devices’ agreement in classification for nocturnal hypertension and dipping patterns, using method-specific measurement schedules.

### Usability

To assess usability, or user-friendliness, we used the system usability scale (SUS) questionnaire [15]. The SUS is a validated 10-item questionnaire on a 5-point Likert scale assessing perceived usability, reflecting how easy, efficient, and satisfactory a system is to use (Table 2, Supplemental Digital Content). Example items include statements such as “I think that I would like to use this system frequently”, “I found the system unnecessarily complex”, and “I thought the system was easy to use”. Participants rated each item from 1 (strongly disagree) to 5 (strongly agree). Items were scored according to standard rules: for positively worded items (1, 3, 5, 7 and 9), 1 was subtracted from the raw score; for negatively worded items (2, 4, 6, 8 and 10), the raw score was subtracted by 5 [15]. Adjusted item scores were summed and multiplied by 2.5 to yield a total SUS score ranging from 0 (worst usability) to 100 (best usability). In addition, the number of performed measurements as a proportion of the number of planned measurements was calculated as another usability indicator.

Participants were instructed to complete the questionnaire once after the 5-day HBPM measurement period and once after the 24-h ABPM measurement period. All questionnaires were completed online without assistance.

### Sleep quality

Sleep quality at baseline was assessed using the SCOPA-SLEEP questionnaire [16]. Participants completed the SCOPA-SLEEP questionnaire assessing nighttime sleep problems (NS; 5 items with 4 response options), day-time sleepiness (DS; 6 items with 4 response options) and overall nighttime sleep quality (1 item on a 7-point scale) over the past month (Table 1, Supplemental Digital Content). SCOPA-SLEEP NS (range 0–15) and DS (range 0–18) scores were computed by summing item responses, with higher scores indicating worse sleep.

In addition, participants rated overall sleep quality on a visual analogue scale (VAS) ranging from 0 (worst sleep quality) to 100 (best sleep quality) at baseline, once after the 5-day HBPM measurement period and once after the 24-h ABPM measurement period.

### Measurement schedules

Daytime and nighttime BP were assessed by HBPM and ABPM in all included participants within a maximum interval of six weeks. The order of device use was determined by randomization and participants decided themselves on which days and nights they would perform the measurements.

For HBPM, the Omron HEM-9601T (NightView) was used, an automatic wrist-type device using the oscillometric technique [12]. The device included a timer function for nighttime BP. Participants were instructed to activate the nocturnal mode before bedtime. Three automated nighttime readings were scheduled each night: 4 h after bedtime, at 02 : 00, and at 04 : 00. For daytime measurements, participants were instructed to take single morning (07 : 00–12 : 00) and evening (18 : 00–23 : 00) measurements after five minutes of rest in the sitting position, with the wrist at heart level. Measurements were performed on five days and nights, not necessarily consecutive.

For ABPM, the Microlife WatchBPO3 device was used, a fully automatic upper-arm device using the oscillometric technique [17]. The monitor was programmed by the study's research nurse, and participants could contact the research team in case of problems. BP was recorded every 60 min for 24-h. Participants were instructed to follow normal daily routines, avoid extremely strenuous activity, and remain still with the forearm extended during measurements.

Participants were trained in device use and were provided written instructions.

HBPM data were exported via the Omron Connect app and ABPM data via a computer link. Analyses were based on data obtained from the device memory.

### Power calculation

Sample size was calculated for a 15-point difference in mean VAS sleep quality scores between HBPM and ABPM devices. With a two-sided alpha of 0.05 and power of 0.8, 28 participants per arm were required. Accounting for 5% dropout, the target sample size was 30 participants per arm (60 participants total).

### Statistical analysis

Continuous data are presented as mean ± standard deviation (SD) unless indicated otherwise. To compare usability (SUS) and VAS-measured sleep quality between devices, linear-mixed-effects (LME) models were fitted using restricted maximum likelihood (REML), including a random intercept for each participant to account for repeated measures in the crossover design. Fixed effects included the device (HBPM versus ABPM), randomization group (HBPM or ABPM first), and their interaction. For sleep quality, baseline SCOPA VAS sleep quality was also included as a covariate. Device × randomization group interactions were tested and removed when nonsignificant. Estimated marginal means (EMMs) with 95% confidence intervals (CIs) and pairwise comparisons between devices were obtained from the final LME models.

BP measurements with systolic BP (SBP) <60 mmHg or >250 mmHg and with diastolic BP (DBP) <30 mmHg or >150 mmHg were considered invalid and were discarded. For HBPM, daytime BP was calculated as the mean of morning and evening measurements, and nighttime BP as the mean of the three nocturnal readings, computed for each of the five nights separately and for the 5-night average. For ABPM, in the absence of objectively recorded sleep-wake times, nighttime was defined according to a narrow-fixed interval (24 : 00–06 : 00) and daytime as 7 : 00–23 : 00, with means calculated from the hourly measurements within each period [3]. Nocturnal hypertension was defined as nighttime SBP ≥120 mmHg and/or DBP ≥70 mmHg, and dipping status as nocturnal BP reduction <10% for SBP and/or DBP, determined for each night separately for both HBPM and ABPM, and for the 5-night HBPM average. A two-sided probability value *P* < 0.05 was considered statistically significant. Analyses were performed using R (version 4.5.2).

## RESULTS

### Participants’ characteristics

A total of 142 participants from the RED-LRGP study were approached, and 62 were willing to participate, of whom 2 dropped-out. Sixty participants completed the questionnaires and were included in the BP analyses (women 56.5%, mean age 58.1 ± 6.8 years, clinic SBP 130.0 ± 15.9 mmHg, clinic DBP 80.3 ± 9.6 mmHg). Fourteen participants (22.6%) had hypertension (Table 1). Thirty of the 60 participants (50%) were randomized to use HBPM first.

**TABLE 1:** Baseline characteristics

Characteristics	
Number of participants	62
Mean age in years (SD)	58.1 (6.8)
Sex	
Female	35 (56.5%)
Male	27 (43.5%)
Mean BMI in kg/m^2^ (SD) (*n* = 59)	27.3 (4.2)
Mean office SBP in mmHg (SD) (*n* = 59)	130.0 (15.9)
Mean office DBP in mmHg (SD)(*n* = 59)	80.3 (9.6)
*Comorbidities*	
Hypertension	14 (22.6%)
Type 2 diabetes	5 (8.1%)
Previous major adverse cardiovascular event (MACE)^*^	0
eGFR <60 ml/min/1.73 m^2^ (*n* = 59)	3 (5.1%)
*Antihypertensive drugs*	
One	9 (14.5%)
Two	0
Three	1 (1.6%)

* MACE is defined as a composite of nonfatal stroke, nonfatal acute myocardial infarction, and cardiovascular death.

### Usability

We observed higher usability scores for HBPM (mean SUS 71.9 (SE 1.4, 95% CI: 69.1–74.7)) as compared to ABPM (mean SUS 52.4 (SE 1.4, 95% CI: 49.6–55.2)). On average, ABPM scored 19.5 points lower than HBPM (95% CI: 15.6–23.4, *P* < 0.0001). The crossover sequence (randomization group) did not influence SUS scores difference between the devices (*P* = 0.36).

**FIGURE 1:**
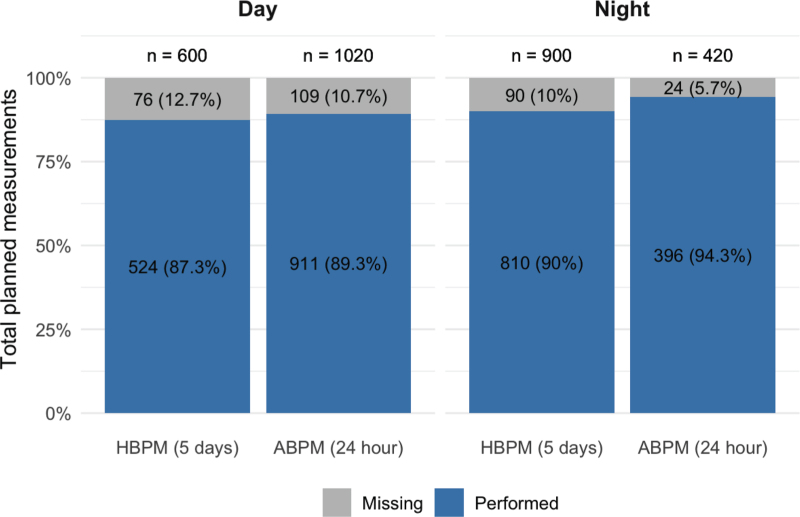
Proportion of planned daytime and nighttime home and ambulatory blood pressure measurements performed. ABPM, ambulatory blood pressure monitoring; HBPM, home blood pressure monitoring. Each bar has been standardised to 100% height to represent the total planned measurements for daytime and nighttime periods for HBPM and ABPM. The total number of planned measurements (n) is indicated above each bar. Each stacked segment shows the number and percentage of performed (blue) and missing (grey) measurements.

In total, 524 out of 600 planned daytime HPBM readings were performed (87.3%; 2 measurements per day for 5 days across 60 participants). For nighttime measurements, 810 out of 900 readings were performed (90.0%; 3 measurements per night for 5 nights across 60 participants). For ABPM, 911 out of 1020 daytime readings (89.3%; 17 measurements across 60 participants) and 396 out of 420 nighttime readings (94.3%; 7 measurements across 60 participants) were performed (Fig. 1). Twenty-five participants (41.6%) had complete HBPM measurements, and 30 participants (50.0%) had complete ABPM measurements. Incomplete data for both devices were primarily due to BP device errors (e.g., motion artifacts, incorrectly applied cuffs). All HBPM readings were valid, whereas 1 daytime and 2 nighttime ABPM readings were invalid (SBP <60 mmHg or >250 mmHg, or DBP <30 mmHg or >150 mmHg).

### Sleep quality

At baseline, participants reported a mean SCOPA-NS score of 9.0 ± 2.8 (range 0–12), and a mean SCOPA-DS score of 8.4 ± 1.9 (range 0–9). The overall nighttime sleep quality score was 3.0 ± 1.2 (range 0–5) and overall sleep VAS score was 75.4 ± 13.6 (range 24–95).

Participants reported significantly higher sleep quality with the wrist-type HBPM compared with the upper-arm ABPM (Table 2). The estimated mean VAS score was 74.6 (SE 2.4, 95% CI: 69.9–79.2) for HBPM and 58.9 (SE 2.4, 95% CI: 54.3–63.6) for ABPM, yielding a mean difference of 15.6 points (95% CI: 9.3–22.0, *P* < 0.0001). When compared with overall baseline sleep quality (VAS 75.4), the mean VAS score declined by 0.8 points with HBPM, whereas ABPM was associated with a 16.5-point decline. The crossover sequence (randomization group) did not influence the difference in VAS scores between the devices (*P* = 0.34).

**TABLE 2:** SUS scores and overall VAS sleep quality after using HBPM versus ABPM device

Outcome	Device	EMM [95% CI]	Standard error	Paired difference [95% CI]	*P*-value
SUS score	HBPM	71.9 [69.1, 74.7]	1.4	19.5 [15.6, 23.4]	<0.0001
	ABPM	52.4 [49.6, 55.2]	1.4		
Overall sleep VAS	HBPM	74.6 [69.9, 79.2]	2.4	15.6 [9.3, 22.0]	<0.0001
	ABPM	58.9 [54.3, 63.6]	2.4		

ABPM, ambulatory blood pressure monitoring; CI, confidence interval; EMM, estimated marginal mean; HBPM, home blood pressure monitoring; SUS, system usability scale; VAS, visual analogue scale. SUS score, sum of 10 items assessing usability of the device (higher scores indicate better usability); overall sleep VAS: 0 = worst sleep, 100 = best sleep.

### Blood pressure measurements

For wrist-type HBPM, mean daytime systolic and diastolic BPs were 126 ± 14 mmHg and 80 ± 10 mmHg, respectively, and mean nighttime systolic and diastolic BPs were 103 ± 13 mmHg and 61 ± 9 mmHg, respectively. For upper-arm ABPM, mean daytime systolic and diastolic BPs were 121 ± 11 mmHg and 74 ± 7 mmHg, respectively, and mean nighttime systolic and diastolic BPs were 108 ± 13 mmHg and 64 ± 9 mmHg, respectively. The BPs measured by HBPM per day, with corresponding time points, are summarized in Table 3, Supplemental Digital Content.

### Detection of nocturnal hypertension and nondippers

**FIGURE 2:**
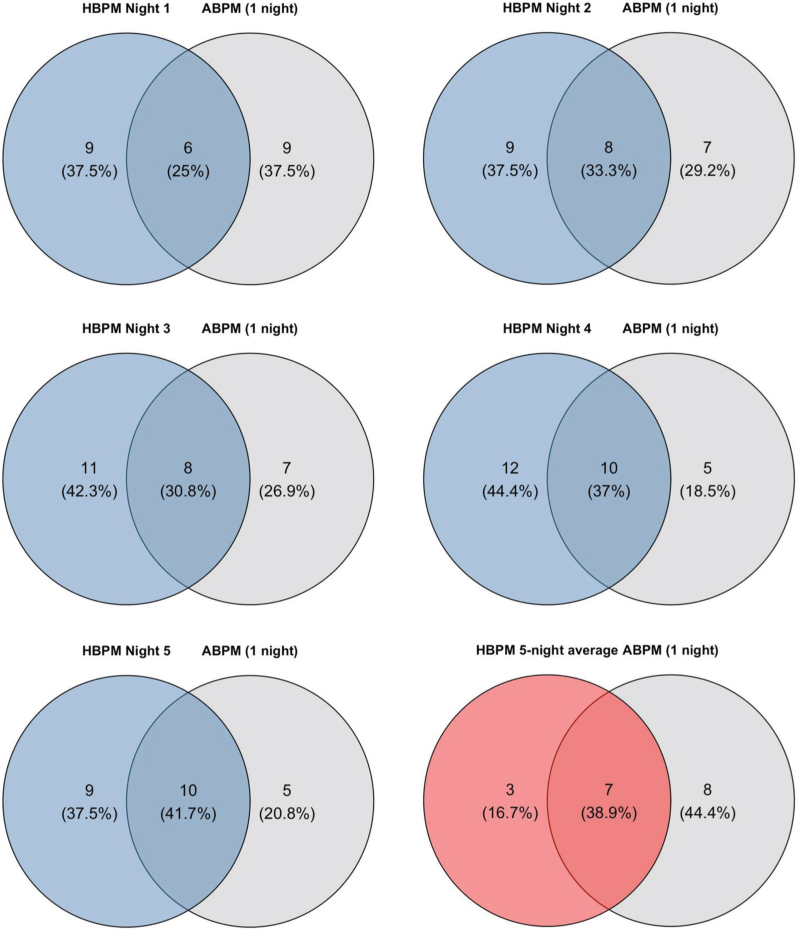
Overlap of nocturnal hypertension detected by 5-night HBPM and single-night ABPM. ABPM, ambulatory blood pressure monitoring; HBPM, home blood pressure monitoring. Diagrams show the number and proportion of participants with nocturnal hypertension detected by HBPM only (per night or 5-night average), ABPM only (single night), or both devices, relative to the total number of participants with nocturnal hypertension.

HBPM detected nocturnal hypertension (nocturnal SBP ≥ 120 mmHg and/or nocturnal DBP ≥ 70 mmHg) in 15/56 (26.8%), 17/57 (29.8%), 19/56 (33.9%), 22/56 (39.3%), and 19/55 (34.5%) participants on nights 1 to 5, respectively, compared with 15/58 participants (25.9%) measured on a single night by ABPM (Figure 1, Supplemental Digital Content). Using the 5-night average HBPM, 10 (16.9%) participants were classified as hypertensive. The proportion of participants in whom nocturnal hypertension was detected by both devices was 25.0% (6 of 24 participants with nocturnal hypertension), 33.3% (8 of 24), 30.8% (8 of 26), 37.0% (10 of 27), and 41.7% (10 of 24) on HBPM nights 1 to 5, respectively, compared with the single night of ABPM (Fig. 2). Using the 5-night average HBPM, this proportion was 38.9% (7 of 18).

**FIGURE 3:**
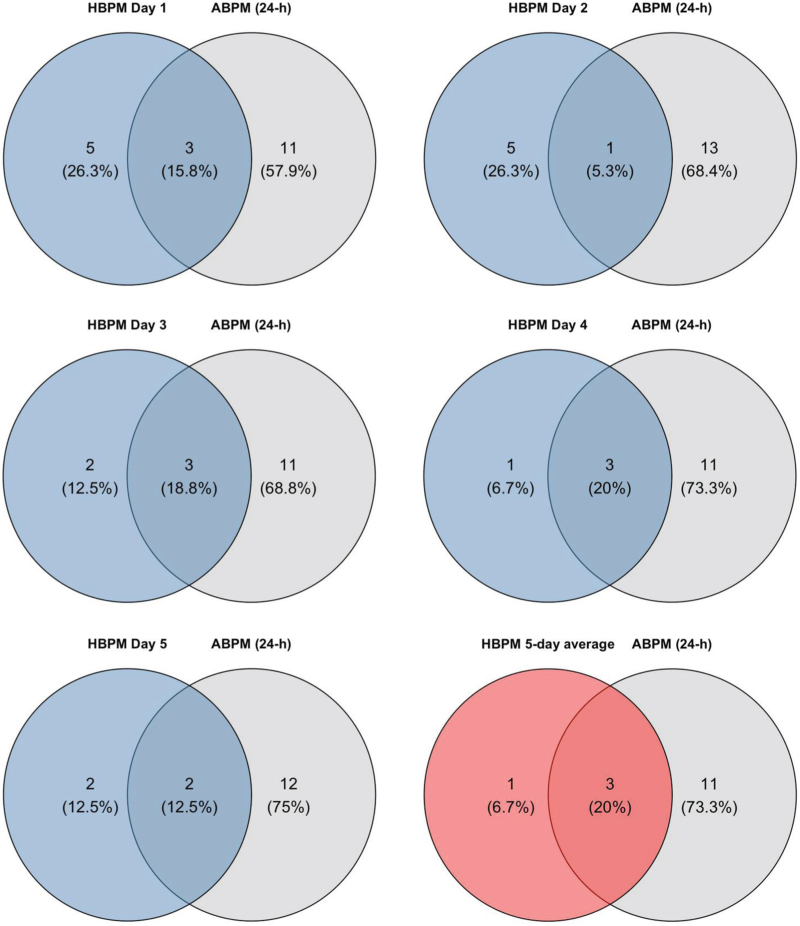
Overlap of nondipper status detected by 5-day HBPM and 24-h ABPM. ABPM, ambulatory blood pressure monitoring; HBPM, home blood pressure monitoring. Diagrams show the number and proportion of participants classified as nondippers by HBPM only (per day or 5-day average), ABPM only (24-h), or both devices, relative to the total number of nondippers on each day.

HBPM identified nondippers (nocturnal dip <10% for SBP and/or DBP measurement) in 8/56 (14.3%), 6/57 (10.5%), 5/55 (9.1%), 4/56 (7.1%) and 4/55 (7.3%) participants on days 1 to 5, respectively, compared with 14/57 participants (24.6%) by ABPM (Figure 2, Supplemental Digital Content). Using the 5-day average HBPM, 4 (6.8%) participants were classified as nondippers. The proportion of nondippers detected by both devices was 15.8% (3 of 19 nondippers), 5.3% (1 of 19), 18.8% (3 of 16), 20.0% (3 of 15), and 12.5% (2 of 16) on HBPM days 1 to 5, respectively, compared with a single 24-h ABPM (Fig. 3). Using the 5-day average HBPM, this proportion was 20.0% (3 of 15).

In total, sixteen participants (mean age 59 years, 69% female) were classified as HPBM nondippers on one or more nights. They had no obesity (body mass index (BMI) <30 kg/m^2^, mean BMI 25.7 kg/m^2^) or other relevant comorbidities such as hypertension, type 2 diabetes, or reduced renal function (eGFR <60 mL/min/1.73 m^2^). In contrast, ABPM detected a nondipping pattern in 14 participants (mean age 60 years, 75% female), including 4 hypertensive patients (one on three antihypertensive drugs) and one with type 2 diabetes, but no obesity (mean BMI 27.9 kg/m^2^).

## DISCUSSION

### Summary

In this randomized crossover study comparing a wrist-type HBPM device, used for 5 days and nights, with a conventional upper-arm 24-h ABPM device, HBPM was rated substantially more user-friendly and caused less sleep disruption. Repeated nocturnal HBPM captured night-to-night BP variability with detection of nocturnal hypertension in 17% of participants using the 5-night average versus 26% by single-night ABPM, and nondipping in 7% versus 25% by ABPM.

### Comparison with existing literature

Our findings support the evidence that patients generally prefer HBPM over ABPM [18–21]. Questionnaire responses indicated that ABPM caused more discomfort, particularly due to restrictions on daily activities and frequent nighttime measurements that could disturb sleep [18,19], although overall sleep quality was reported to be similar [19]. In contrast, in our study, participants reported significantly higher sleep quality with HBPM (VAS 74.6) compared to ABPM (VAS 58.9), likely due to fewer nightly measurements and better comfort with a wrist-type device than with an upper-arm device. When compared with baseline sleep quality (VAS 75.4), scores with HBPM were essentially unchanged, whereas ABPM was associated with a marked decline. In addition, studies involving more than 50 hypertensive patients using the same wrist-type device as in our study (Omron HEM-9601T) also reported less sleep disturbance and discomfort compared with upper-arm monitoring [13,22].

Our finding that multinight wrist-type HBPM detected nocturnal hypertension in a slightly higher proportion of participants on individual nights (27–39%) than single-night upper-arm ABPM (26%) aligns with evidence of limited reproducibility of nighttime BP based on a few measurements, regardless of assessment method [6]. Similarly, a wrist-type HBPM study in 46 hypertensive patients measuring nocturnal BP over two consecutive nights, demonstrated considerable night-to-night variability [23]. Averaging measurements across five HBPM nights reduced the proportion classified as hypertensive, likely by mitigating random variability and regression to the mean, resulting in a more stable estimate of usual nocturnal BP.

In our study, wrist-type HBPM also showed notable night-to-night variability in dipping status, with 7% to 14% classified as nondippers on individual nights versus 7% based on the 5-day average. This aligns with the J-HOP Nocturnal BP study in 927 participants, which showed that single-night dipping status often differed from multinight home BP assessment [24]. Observed variability in our study likely reflects night-to-night BP fluctuations, influenced by factors such as sleep quality, as even minor nightly BP changes can alter classification when using a dichotomous dipper/nondipper system [25]. In addition, measurement artefacts from daytime movements or body position may contribute to variability [3].

### Strengths and limitations

A major strength of our study is the randomized cross-over design, which allows each participant to serve as their own controls. This approach minimizes the influence of inter-individual variability, such as differences in device experience and habitual sleep quality, on assessments of usability and sleep quality. Moreover, the study included a detailed quantitative assessment of device usability (SUS) and sleep quality (VAS), providing a more comprehensive and continuous evaluation compared to the categorical questions used in previous studies [13,22]. The single assessment for each device reflected participants’ overall impression of sleep quality, reducing the impact of nightly variations. In addition, multinight HBPM measurements, together with systematic recording of performed and missed BP readings, offered a reliable assessment of night-to-night variability in nocturnal BP and dipping patterns, while also providing realistic insight into device usability and impact on sleep quality in daily life.

However, the study also has several limitations. First, differences in measurement schedules, that is, HBPM over five nonfixed days and nights versus single 24-h ABPM, with measurements performed at different times and frequencies, limit comparisons of the proportion of participants with nocturnal hypertension and nondipping patterns detected by both devices, and preclude an exact assessment of agreement between the methods. Nevertheless, these schedules are device-specific and guideline-based [1,26], reflecting their intended use in everyday practice and supporting our primary aim of evaluating usability and impact on sleep quality. Moreover, daytime home BP was measured only once in the morning and evening per day, whereas duplicate measurements for at least three days are recommended [1]. Nevertheless, collecting data over five days likely improved the precision of mean daytime BP estimates. Second, no gold standard exists for assessing dipping status, and there is currently no guideline-endorsed definition for HBPM dipping. Therefore, we used the same threshold for both monitoring methods, and our analyses of agreement in classification between HBPM and ABPM for dipping patterns were exploratory, rather than intended to determine which device performs “better”. Third, participants did not report their sleep periods during ABPM, so it is uncertain whether wake and sleep periods were identified accurately. However, using a narrow fixed interval (24 : 00–06 : 00) may more accurately capture sleep periods than wide intervals (e.g., 23 : 00–07 : 00) [3]. The average bedtime over five HBPM nights was 23 : 18, supporting 24 : 00 as a reasonable cutoff for nighttime BP in ABPM, particularly since the same participants underwent both methods in the cross-over design. In addition, although nocturia was not measured and may increase absolute nocturnal BP, it is unlikely to affect comparisons between HBPM and ABPM, as participants served as their own controls. Last, the study population consisted of mostly middle-aged, relatively healthy participants, limiting generalizability to older or higher-risk populations.

### Implications for practice

The wrist-type HBPM device (Omron HEM-9601T) represents a novel, patient-centered approach to nocturnal BP monitoring. It enables automatic, multinight home BP measurements with high usability and minimal sleep disturbance. Its integrated timer function and companion app eliminate the need for trained personnel or clinic-based equipment, making long-term monitoring more practical and patient-friendly than conventional upper-arm ABPM. In clinical practice, wrist-type HBPM may improve patient adherence and facilitate more comprehensive assessment of nocturnal BP patterns, offering a feasible alternative to conventional upper-arm ABPM.

Importantly, the observed night-to-night variability in HBPM-derived nocturnal hypertension and dipping status illustrates the limitation of relying on single-night measurements to classify individual's nocturnal BP patterns. Repeated nocturnal measurements may provide a more reliable and nuanced characterization of an individual's nocturnal BP profile. Future work should establish consensus definitions or guideline recommendations for HBPM-derived dipping status, and explore method-specific agreement with ABPM under standardized measurement windows. Integrating sleep and nocturia assessments may further clarify the determinants of nocturnal BP variability and inform both usability and clinical interpretation.

## CONCLUSION

Wrist-type HBPM, combining multinight automated monitoring with high usability and minimal sleep disruption represents a feasible, patient-centered tool for home-based nocturnal BP assessment that has the potential to capture night-to-night BP variability by measuring across multiple nights.

## ACKNOWLEDGEMENTS

No acknowledgements.

### Conflicts of interest

OMRON Healthcare Europe B.V. provided financial support and access to the investigational device, and had no role in the study design, data collection, analysis, interpretation, or the decision to publish. All authors declare no conflict of interest.

## Supplementary Material

Supplemental Digital Content
