# Supplementary material for: Wrist-type home blood pressure monitoring device improves usability and preserves sleep quality compared with conventional ambulatory monitoring
Source: J Hypertens. 2026 Mar 20;44(6):1032–40. doi: 10.1097/HJH.0000000000004297 (PMC13152039; doi:10.1097/HJH.0000000000004297)
Supplement: Supplemental Digital Content [file jhype-44-1032-s001.pdf]

Supplementary

Table 1. SCOPA-SLEEP questionnaire

Questions	Response categories
**A. Use of sleeping tablets**	**Not at all – less than once a week – once or twice a week – more than 3 times a week**
A1. How often did you use sleeping tablets in the last months? (prescribed by a physician or not)	
A2. Which sleeping tablets did you use in the last month? [name; amount per month; dose per tablet]	
**B. NS: Nighttime sleep problems**	**Not at all – a little – quite a bit – a lot**
B1. In the past month, have you had trouble falling asleep when you went to bed at night?	
B2. In the past month, to what extent do you feel that you have woken too often?	
B3. In the past month, to what extent do you feel that you have been lying awake for too long at night?	
B4. In the past month, to what extent do you feel that you have woken up too early in the morning?	
B5. In the past month, to what extent do you feel you have had too little sleep at night?	
**C. Overall sleep quality**	**Very well – well – rather well – not well but not badly – rather badly – badly – very badly**
**DS: Day-time sleepiness**	**Never – sometimes – regularly – often**
D1. How often in the past month have you fallen asleep unexpectedly either during the day or in the evening?	
D2. How often in the past month have you fallen asleep while sitting peacefully?	
D3. How often in the past month have you fallen asleep while watching TV or reading?	
D4. How often in the past month have you fallen asleep while talking to someone?	
D5. In the past month, have you had trouble staying awake during the day or in the evening?	
D6. In the past month, have you experienced falling asleep during the day as a problem?	

Table 2. System Usability Score questionnaire

Questions	Strongly disagree to strongly agree				
	**1**	**2**	**3**	**4**	**5**
1. I think that I would like to use this system* frequently					
2. I found the system unnecessarily complex					
3. I thought the system easy to use					
4. I think that I would need the support of a technical person to be able to use this system					
5. I found the various functions in this system were well integrated					
6. I thought there was too much inconsistency in this system					
7. I thought there was too much inconsistency in this system					
8. I found the system very cumbersome to use					
9. I fel very confident using the system					
10. I needed to learn a lot of things before I could get going with this system					

*System was adapted to blood pressure monitor.

Table 3. Average daytime and nighttime blood pressure measurements for HBPM device, shown per time point and per day

Day	Measurement	Time point (hh:mm)	Systolic blood pressure in mmHg (SD)	Diastolic blood pressure in mmHg (SD)
1	Morning	07:35	122 ± 17	78 ± 14
	Evening	19:08	131 ± 20	82 ± 14
	Night: 4h after bedtime	03:12	101 ± 15	59 ± 10
	Night: scheduled	02:00	103 ± 16	60 ± 10
	Night: scheduled	04:00	104 ± 14	61 ± 10
2	Morning	07:39	119 ± 16	77 ±13
	Evening	18:52	129 ± 18	82 ± 14
	Night: 4h after bedtime	3:18	100 ± 12	59 ± 7
	Night: scheduled	02:00	102 ± 14	61 ± 9
	Night: scheduled	04:00	103 ± 14	60 ± 10
3	Morning	08:18	125 ± 18	79 ± 14
	Evening	18:29	127 ± 18	79 ±12
	Night: 4h after bedtime	03:22	101 ± 16	60 ± 11
	Night: scheduled	02:00	103 ± 17	60 ± 11
	Night: scheduled	04:00	104 ± 15	60 ± 10
4	Morning	07:25	127 ± 16	79 ± 12
	Evening	18:47	129 ± 17	80 ± 12
	Night: 4h after bedtime	03:18	102 ± 14	60 ± 10
	Night: scheduled	02:00	102 ± 16	60 ± 10
	Night: scheduled	04:00	104 ± 16	60 ± 10
5	Morning	07:38	123 ± 18	75 ± 14
	Evening	18:04	129 ± 16	80 ± 12
	Night: 4h after bedtime	03:19	102 ± 17	61 ± 11
	Night: scheduled	02:00	102 ± 16	60 ± 12
	Night: scheduled	04:00	102 ± 14	59 ± 10

Abbreviations: HBPM = home blood pressure monitoring.

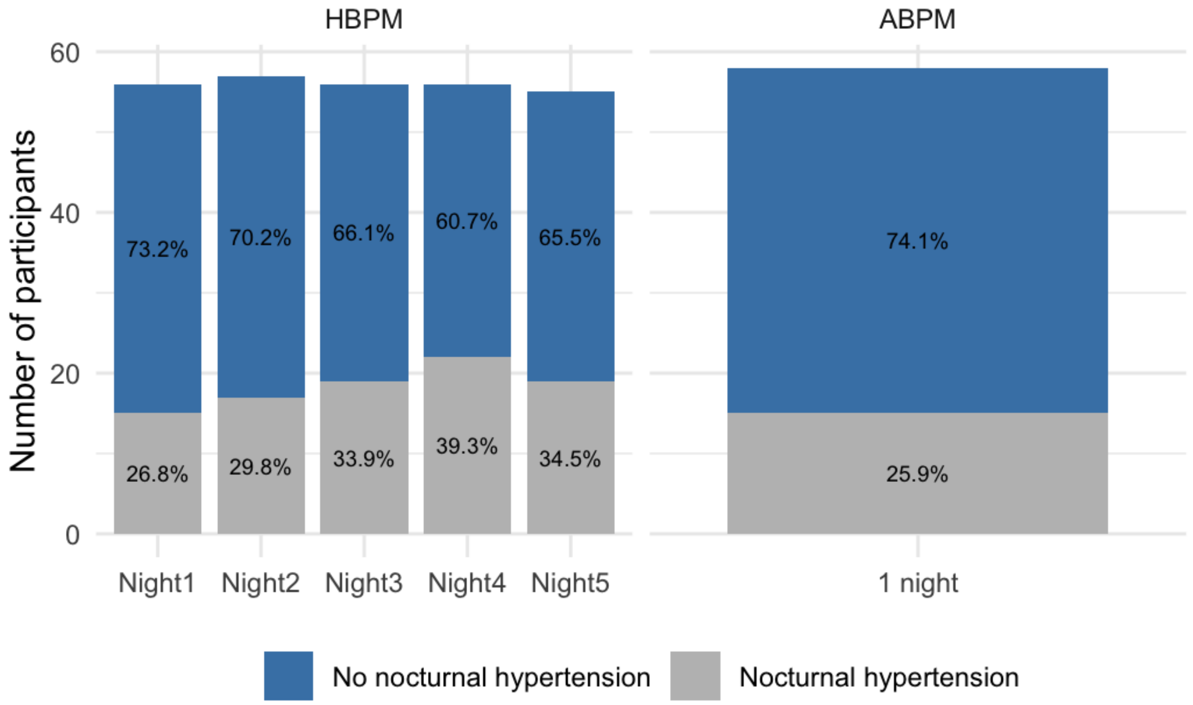

Figure 1. Nocturnal hypertension detected by HBPM on nights 1-5 and by a single night ABPM

Abbreviations: HBPM = home blood pressure monitoring; ABPM = ambulatory blood pressure monitoring.

Bar charts show the proportion of participants without nocturnal hypertension (blue) versus with nocturnal hypertension (grey) for HBPM nights 1-5 and 24-h ABPM.

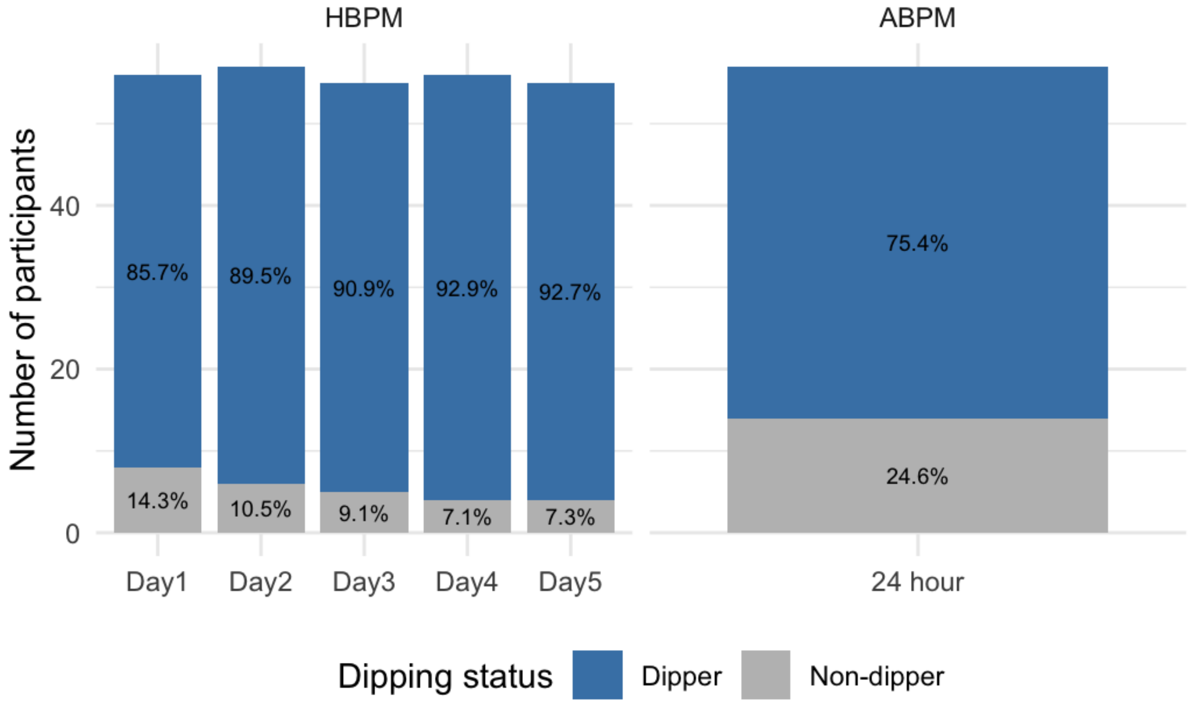

Figure 2. Dipping status detected by HBPM on days 1-5 and by 24-h ABPM

Abbreviations: HBPM = home blood pressure monitoring; ABPM = ambulatory blood pressure monitoring.

Bar charts show the proportion of dippers (blue) versus non-dippers (grey) for HBPM days 1-5 and 24-h ABPM.
